# Physiological adaptation after a 12-week physical activity program for patients with Prader–Willi syndrome: two case reports

**DOI:** 10.1186/s13256-016-0966-8

**Published:** 2016-06-23

**Authors:** Alexandre Slowetzky Amaro, Maria Cristina Triguero Veloz Teixeira, Maria Luiza Guedes de Mesquita, Graciele Massoli Rodrigues, Daniela Andrea Rubin, Luiz Renato Rodrigues Carreiro

**Affiliations:** 1 Programa de Pós-graduação em Distúrbios do Desenvolvimento, Universidade Presbiteriana Mackenzie, Rua da Consolação, 930, São Paulo, SP CEP - 01302-907 Brasil; 2 Escola Superior de Educação Física de Jundiaí, Rua Dr. Rodrigo Soares de Oliveira, s/n - Anhangabau, Jundiaí, SP CEP - 13208-120 Brasil; 3 Programa de Pós-Graduação Stricto Sensu em Ciências do Envelhecimento, Universidade São Judas Tadeu, Rua Taquari, 546, São Paulo, SP CEP 03166-000 Brasil; 4 Department of Kinesiology, California State University Fullerton, 800 N. State College Blvd, Fullerton, CA 92834 USA

**Keywords:** Physical activity, Prader–Willi syndrome, Physical fitness and body composition

## Abstract

**Background:**

Physical activity programs are a powerful tool against several diseases including obesity and their comorbidities. Prader–Willi syndrome is the most common genetic disease associated with obesity, and brings with it behavioral and emotional problems that need complex management. Research into the effect of physical activity programs on Prader–Willi syndrome is limited and it is frequently argued that if a physical activity program is too complex, the participants are more likely to drop out. Therefore, in this study, we assessed the physiological adaptation effect of a physical activity program with increasing complexity and load, in a boy and a girl with Prader–Willi syndrome by assessing changes in lipid profile, body composition, and physical fitness parameters.

**Case presentation:**

Case 1 was an 11-year-old girl, mixed race (brown), with an intelligence quotient of 68, 52.0 % body fat, and a body mass index of 45.3 kg/m^2^. The Prader–Willi syndrome diagnosis was made when she was 5-years old and was found to be due to an imprinting genomic defect. Case 2 was a 14-year-old boy, mixed race (brown), with an intelligence quotient of 74, 48.8 % body fat, and a body mass index of 37.3 kg/m^2^. The diagnosis was made when he was 10-years old and was found to be caused by gene deletion. Both participants presented physical characteristics and behavior problems typical of Prader–Willi syndrome. Case 2 presented high blood pressure, high cholesterol and sleep apnea and had to use continuous positive airway pressure to sleep. Both participants were assessed for 12 weeks (three times a week) using a physical activity program designed to improve strength and muscle hypertrophy. The work load was progressively adjusted as necessary and new exercises were added to the program. Prior to the program, the participants’ parents received instructions about managing problem behavior and advice about nutrition.

**Conclusions:**

After physical activity program several health markers assessed by biological tests and parental report had improved in both participants. The participants positively accepted the adaptations made to the physical activity program during the study. More studies are necessary to assess the benefits of physical activity in the Prader–Willi syndrome population.

## Background

Prader–Willi syndrome (PWS) is the genetic disorder most frequently associated with obesity. People with PWS do not experience a feeling of satiety, even postprandial, due to hypothalamic abnormality [1]. Endocrine and metabolic alterations such as growth hormone (GH) deficit and gonadotrophic hormone deficit effectively contribute to the development of obesity and metabolic syndrome [2]. PWS is also marked by cognitive difficulties such as intellectual disability and executive function deficits [3]. Ritualistic behavior and explosive outbreaks of anger, particularly when the individual’s routine is broken, are common [4, 5]. All these associated conditions contribute to lower levels of physical activity and/or adherence to physical activity programs (PAP) [6]. Among the studies that report the effects of PAPs in PWS, most of them comprise unchanged routines throughout the program [7–10]. It is argued that increasing task complexity and the physical stress caused by the exercise could lead to the participants dropping out of the training programs [11]. However, interventions designed with unchanging activities can limit physiological adaption [12, 13]. Therefore, the present study assessed the physiological adaptation effect of a PAP with increasing complexity and workload in a boy and a girl with PWS by measuring lipid profile, body composition, and physical fitness parameters. This study was approved by the Research Ethics Committee of Mackenzie Presbyterian University (CEP/UPM N° 1432/04/2012).

### Assessment and instruments

For cognitive and behavioral characterization, an estimate intelligence quotient (IQ) score was obtained through the Wechsler Intelligence Scale for Children – third edition (WISC-III) subtests Block Design and Vocabulary [14] and the Child Behavior Checklist for ages 6 to 18 (CBCL/6–18) [15]. Anthropometrics data from participants were measured and their body mass indices (BMI) were calculated according to the World Health Organization (WHO) child growth standards [16]. A dual-energy X-ray absorptiometry scan (Discovery Wi – S/N 84206, Hologic Inc.) was used to assess body composition parameters such as percentage of body fat, lean mass, bone mineral density (BMD), and bone mineral content (BMC) of the lumbar spine and body total [17]. A battery of measures and somatomotor tests called PRODOWN were administered to obtain flexibility, strength, explosive strength, agility, displacement velocity, and cardiorespiratory endurance measurements [18]. In addition, daily physical activity was measured using pedometers and a physical activity level questionnaire (PALQ) [9]. Fasting blood analyses was conducted to obtain a lipid, glycemia, and uric acid profile. A complete description of items for each assessment is included in Table 1.

**Table 1:** Pre- and post-intervention assessment types and their respective instruments

Assessment	Rate/Tool
Wechsler Intelligence Scale for Children – third edition	Subtests Block Design and Vocabulary
Children Behavior Checklist 6–18 years	Questionnaire
Anthropometrics	Weight
	Height
	Body mass index
Body composition	Dual-energy X-ray absorptiometry used for assessing total and lumbar bone mineral density, bone mineral content, body fat rate, and lean mass
PRODOWN battery of measures and somatomotor tests	Flexibility test (sit and reach test)
	Abdominal strength endurance test
	Lower limbs explosive strength test (standing long jump)
	Upper limbs explosive strength test (medicine ball throw)
	Agility test (square test)
	Displacement speed test (20-meter run)
	Cardiorespiratory capacity test (6 minutes)
Physical activity level	Physical activity level questionnaire
	Spontaneous physical activity (pedometer)
Blood parameters	Standard protocols for blood measures (cholesterol, glycemia, triglycerides, and uric acid)

### PAP

The PAP was based on tasks successfully used in previous studies on PWS [9, 10]. The complete program was composed of 36 sessions of 60 minutes delivered over 12 weeks (Table 2). Every 3 weeks, a new task was introduced and loads were adjusted. The proposed exercises were: ankle dorsiflexion on platform, hip extension, bench squat, medicine ball chest pass, overhead medicine ball catch and throw, ball games, 40-meter run, and playful activities with ball and balloon.

**Table 2:** Training program according to phase, duration, and type of training

Phase	Duration	Type of training
1	3 weeks (3 times a week)	Muscular Endurance 1: ankle flexion and extension, throw a 2-kg medicine ball Coordination: keep a balloon suspended by using different body parts and objects Aerobic: 15-minute walk
2	3 weeks (3 times a week)	Muscular Endurance 1: as in phase 1, but with a 3-kg medicine ball Muscular Endurance 2: hip elevation, catch and hold a 2-kg medicine ball as it bounces from the floor Coordination: bounce the ball, throw it up and hold Aerobic: 30-minute walk
3	3 weeks (3 times a week)	Muscular Endurance 1: as in phase 2 Muscular Endurance 2: as in phase 2, but with a 3-kg medicine ball Muscular Endurance 3: stand up and sit on a bench Coordination: bounce a ball with alternate hands, throw up and hold with only one hand Aerobic: 30-minute walk
4	3 weeks (3 times a week)	Muscular Endurance 1: as in phase 3 Muscular Endurance 2: as in phase 3 Muscular Endurance 3: as in phase 3 Muscular Endurance 4: go up and down one step Coordination: bounce a ball with alternate hands, throw up and hold with only one hand, with alternate hand for throw and reception

## Case presentation

### Case 1

Case 1 was an 11-year-old girl with a cytogenetic diagnosis of PWS due to mutation in the imprinting center. The diagnosis was confirmed when she was 5 years and 8 months old. She presented intellectual disability (IQ=68) and the main behavioral problems reported by her parents were: not being very active, feeling tired without reason, being too dependent, having poor motor coordination, skin picking, not following rules, being stubborn, and screaming a lot. She lived with her father, stepmother, and two older siblings; she was enrolled in the fourth grade of a mainstream elementary school. She had poor reading and writing skills. Her parents reported that despite their frequent requests for the school to control her food intake no action had been taken and after 5 months she had gained 10 kg.

During the pre-intervention assessment, her parents reported that she was not very active and spent most of the time sitting, watching TV or playing with her dolls. She walked with difficulty when she had to cover longer distances and often stopped to rest.

During the initial assessment, she remained quiet, listening attentively to the orientations for the tasks and demonstrated interest and willingness to start the PAP. Her parents received orientation from a nutritionist trained in PWS who offered menu recommendations adapted to the disorder. They also attended workshops held by a psychologist who gave advice about phenotypic behavioral characteristics and the behavioral management of PWS.

After considering her availability, it was decided to carry out the PAP at our university twice a week and at her house once a week. She accepted positively all the PAP routines, and most of the time the inclusion of new exercises. In the face of new challenges, she understood that they should be overcome. A higher number of repetitions or changing to a heavier medicine ball was also almost always well accepted. If she complained about some change in the PAP, the instructor explained that the alteration was important for her health. She seemed to understand and the session continued. The increase in the number of repetitions was the main cause of complaints. However, the inclusion of games was a motivator to complete tasks. She was praised after completing set tasks to reinforce the behavior. In the final assessment, her parents reported that before the beginning of the PAP she used to have nocturnal enuresis, which ceased after the 12 weeks of training. She attended 95 % of the sessions. Table 3 presents anthropometric and body composition results pre- and post-intervention. There were no initial results for uric acid and glucose for her, but after the PAP they were found to be at normal levels for sex and age (79 and 61.1 mg/dL respectively). In addition, she presented a reduction in total cholesterol (pre=166; post=159 mg/dL), very low-density lipoprotein (VLDL; pre=16; post=14 mg/dL), high-density lipoprotein (HDL; pre=42; post=33 mg/dL), and triglyceride (pre=82; post=72 md/dL), and a small increase in low-density lipoprotein (LDL; pre=109; post=112 mg/dL).

**Table 3:** Anthropometric and body composition data

Assessed item	Participants					
	Case 1			Case 2		
	Data		Variation	Data		Variation
	Pre	Post		Pre	Post	
Height (cm)	136.0	138.0	+2.0	151.0	151.0	0
Total body mass (g)	84,056.8	80,733.8	−3323.0	85,168.4	88,147.7	+2979.3
BMI (kg/m^2^)	45.3	42.5	−2.8	37.3	38.7	+1.4
Fat mass (g)	43,712.2	40,726.4	−2985.8	41,568.5	45,265.2	+3696.7
Lean mass (g)	39,358.7	38,996.3	−362.4	42,094.1	41,286.9	−807.2
Body fat (%)	52.0	50.4	−1.6	48.8	51.4	+2.6
Total BMC (g)	985.9	1011.1	+25.2	1505.8	1595.5	+89.7
Total BMD (g/cm^2^)	0.874	0.877	+0.003	0.873	0.908	+0.035
Total z-score	0.0	−0.2	−0.2	−2.1	−1.7	+0.4
Spinal BMC (g)	24.79	25.30	+0.51	32.71	32.88	+0.17
Spinal BMD (g/cm^2^)	0.671	0.714	+0.043	0.747	0.757	+0.010
Spinal z-score	−0.3	−0.1	+0.2	−0.8	−0.9	−0.1

*BMC* bone mineral content, *BMD* Bone mineral density, *BMI* body mass index

### Case 2

Case 2 was a 14-year-old boy with a cytogenetic diagnosis of PWS due to a gene deletion. The cytogenetic test was carried out when he was 10 years and 10 months old. He attended ninth grade elementary school; he had poor writing and reading skills. He had no history of fracture or surgery, but displayed some typical alterations such as myopia. He had minor health problems, such as rhinitis and sinusitis, and other health problems that require special care such as hypercholesterolemia, hypertension, fatty liver disease, and sleep apnea syndrome. He used a continuous positive airway pressure (CPAP) apparatus to facilitate air flow through his upper airway. From the first moment, he demonstrated interest in taking part in the PAP. Such interest was observed in his effort to take the initial assessment tests. He also demonstrated good physical condition and willingness, so that by the third week he was able to perform the complete set of tasks. From the fourth week, three repetitions of 5×40 meters run were added, as well as 5-meter anteroposterior and lateral exercises in a sand box. Whenever he demonstrated being tired or irritable, these exercises were reduced to one repetition. On Saturdays, training sessions took place on a street near his house. It is noteworthy that the street has a 30° angle incline and that the running practice happened in the ascending direction. He sometimes complained during the interval between repetitions; however, he accomplished all tasks after being encouraged. In order to provide new challenges, from the fifth week, a bonus activity was implemented. The activity consisted of a continuous run with increasing distance every week; it started with 60 meters and reached 200 meters in the last week. He also attended 95 % of the sessions.

A positive aspect of the PAP was the participants’ willingness to perform tasks and their frequent request to continue them. This fact can be associated with the introduction of playful elements and the constant encouragement offered during the program. Table 3 presents anthropometric and body composition results pre- and post-intervention. Case 2’s blood test results after the PAP presented an improvement in important health indicators, reaching health-related values for sex and age for uric acid (pre=7.2; post=5.5 mg/dL), total cholesterol (pre=235; post=182 mg/dL), LDL (pre=153; post=119 mg/dL), VLDL (pre=29; post=19 mg/dL), and triglyceride (pre=143; post=95 mg/dL). An increase in glucose (pre=87; post=115 mg/dL) and a reduction in HDL (pre=53; post=44 mg/dL) were also observed.

Table 4 describes the results of PRODOWN physical fitness assessment tests and level of daily physical activity (pedometer and PALQ). Both participants improved their performance in the post-intervention test of upper limb muscle power, agility and 20-meter displacement speed. The female participant (Case 1) demonstrated an improvement in the lower limb power test. The male participant (Case 2) also presented an improved level of physical activity, with a higher number of steps and distance covered, as well as an increase in PALQ total score. For the 6-minute run test, there was little variation for the female participant and a reduction in performance for the male participant.

**Table 4:** PRODOWN physical fitness and Level of Daily Physical Activity test results

Protocol/Assessed item		Participants					
		Case 1			Case 2		
		Assessment		Variation	Assessment		Variation
		Pre	Post		Pre	Post	
PRODOWN physical fitness	Flexibility sit and reach (cm)	27	27	0	0	2	+2
	Abdominal endurance	0	0	0	0	0	0
	Lower muscles power (cm)	10	24.2	+14.2	76	63	−13
	Upper muscles power (cm)	177	200	+23	275	310	+35
	Square agility (seconds)	15:25	13:39	−1:86	9:30	9:20	−0:10
	20-m displacement speed (seconds)	11:56	11:03	−0:53	6:60	6:30	−0:30
	6-minute run (m)	400	395	−5	580	507	−73
Pedometer	Steps	15,423	10,825	−4595	24,529	31,345	+6816
	Distance (m)	6939	5414	−1525	15,942	20,374	+2865
PALQ	Score	211	199	−12	231	310	+79

Sum of 3 days. Daily recommendation >10,000 steps. *PALQ* physical activity level questionnaire

## Discussion

The participants presented characteristic PWS phenotypic features [19] and both had late cytogenetic diagnosis and were not taking recombinant human growth hormone (rhGH). This might be responsible for both participants presenting such high levels of body fat (~50 %) and short stature at baseline.

During the 12 weeks of the PAP, the female participant grew in height while losing body mass (90 % consisted of fat mass). This result is similar to other studies and demonstrates the positive effect of physical activity for the reduction of body fat [9, 10]. In contrast, the male participant had an increase in body mass, mainly because of an increase in body fat. The lack of supervision of his food intake seems likely to be the main reason for the increase in body fat. Regarding bone status, an increase was observed in lumbar BMD for the female participant and total body BMD for the male participant after the PAP. Before the program, the male participant presented low total body BMD (z-score is −2.1) and after the PAP, the value achieved normal range (z-score is −1.7). According to Duran and colleagues [20], there is a positive correlation between higher levels of physical activity and bone density in PWS. It is possible that the increased physical activity due to PAP contributed to the improvement of the BMD in both participants.

Unlike nonsyndromic obesity, overweight in PWS is not accompanied by an increase in lean mass. Low lean mass and high fat mass could contribute to low levels of physical activity in PWS. In the present study, the female participant displayed a low level of physical activity as assessed by steps/day and questionnaire, while the male participant had a moderate level. Even with a reduction in the level of daily activity measured by the pedometer, the physical educator who implemented the PAP perceived that she improved her willingness and readiness for the accomplishment of daily activities, and was less fatigued during walking. In contrast, the male participant showed an increased level of physical activity, reaching healthy values of over 30,000 steps/day.

In the PRODOWN test, both participants presented improved results for almost all measured variables. However, in the 6-minute test, both participants presented less distance covered after the PAP compared to the initial assessment. Similarly, the male participant did not improve lower limb power. When considering the reasons as to why no improvements were observed in the 6-minute test, it is possible to consider that the participants did not provide their maximum effort. Although the female participant’s data in blood analysis is fragmented, it was possible to observe positive changes in her cholesterolemic profile. This result is consonant with current literature concerning the effects of physical activity on an individual’s blood profile. Although the male participant had an increase in body fat rate, his blood markers indicated an improvement in indexes related to coronary diseases such as LDL, VLDL, triglyceride, and total cholesterol. Their body weight was checked every week and fluctuations during the PAP were observed. In the case of the male participant, such variations may be related to his parents’ difficulties in controlling his access to food.

Energy balance management is the most important task and the highest concern among physicians and parents in the PWS population. Beside the control of energy intake, introducing physical activity into the daily routine is the best non-pharmacologic strategy to maintain healthy weight and prevent obesity. Although patients with PWS are acknowledged to have behavior problems caused by breaks to routines, in this case report the introduction of PAP and the additions made to it over the period of the study did not affect the participants’ moods, even during strenuous exercises. This study was the first to report the effect of PAP on the behavior of patients with PWS. Future studies with a larger sample should investigate how physical activity may affect behavior in patients with PWS.

The key limitations of this study were the small number of participants and the relatively short period of implementation of the program. A longer program would be necessary for the effects of physical activity to be more evident.

Introducing PAP was challenging due to the complexity of PWS. Constant adaptations were required to meet the participants’ needs and the dynamics of each family. As reported in the current literature and observed here, interdisciplinary actions are essential for the success of intervention practices in PWS [10, 21]. This study observed that parental support from psychology, psychiatry and nutrition professionals was essential for the PAP to succeed.

## Conclusions

The completion of this PAP resulted in positive effects in both participants with PWS. Several physical fitness indicators improved, such as body fat reduction, agility, and upper/lower muscle power for the female participant, increased spontaneous physical activity for the male participant and an improvement in upper muscle power. Both participants showed improvement in blood health parameters as well as in bone density. Despite the PAP being successful in improving fitness and health parameters without causing behavioral problems, there is need for constant and multi-professional family support as provided in this intervention.
